# The Influence of Placebo Effect on Craving and Cognitive Performance in Alcohol, Caffeine, or Nicotine Consumers: A Systematic Review

**DOI:** 10.3389/fpsyt.2020.00849

**Published:** 2020-08-26

**Authors:** María Nerea Galindo, José Francisco Navarro, María Cavas

**Affiliations:** Department of Psychobiology, School of Psychology, University of Málaga, Málaga, Spain

**Keywords:** placebo effect, expectancies, craving, cognitive performance, psychoactive substance

## Abstract

**Objective:**

The present systematic review aims to analyze the evidence about the influence of placebo effect on craving and cognitive performance in alcohol, caffeine, and nicotine consumers.

**Methods:**

Relevant studies were identified *via* Pubmed, Web of Science, and Scopus databases (up to March 2020). Only those papers published between 2009 and 2019 were searched.

**Results:**

Of the 115 preliminary papers, 8 studies of database search and 9 of the manual search were finally included in this review. Findings showed that while alcohol expectancies increased craving, caffeine and nicotine expectancies tend to decrease it. Alcohol expectancies caused similar or slower reaction time when alcohol was not consumed, impairments on inhibitory control (especially after alcohol consumption) and similar post-error slowing. The effect of caffeine and nicotine on reaction time has not been elucidated yet, however, caffeine expectancies have been shown to improve accuracy and the attentional filtering of distracting stimuli.

**Conclusions:**

Alcohol, caffeine, and nicotine expectancies play an important role on craving. Although expectancies produce an effect on cognitive performance, caffeine and nicotine beliefs show an ambiguous impact on reaction time. Only the influence of alcohol expectancies on reaction time has been clarified. Furthermore, caffeine beliefs enhance accuracy.

## Introduction

Placebo effect is produced when a treatment without therapeutic characteristics causes a beneficial outcome in the organism (1). Sometimes these procedures provoke an aversive response, which has been denominated nocebo effect (2).

Traditionally, placebo effect has been considered a strange variable to control in investigation. However, researchers began to be interested in this effect in the middle of 20th century. It is important to underline the influence of Beecher’s work (3) since he found that placebo causes a notable improvement in pain symptoms. At that moment placebo effect was considered a potential variable to improve the results of patients, thus, scientists began to investigate the mechanisms underlying this response (2).

Colloca and Miller (4) proposed that placebo effect is a learning response which is produced when individuals generate some expectancies based on contextual, social or treatment cues, and verbal suggestion. Regarding prior experience with that procedure, studies show that a higher number of experiences with the treatment induces a greater placebo response (5). If the experience occurs with a noneffective treatment, placebo effect will be mitigated (6).

Expectancies need learning, which is produced by Pavlovian or instrumental conditioning, suggestion, and social learning (2). On the one hand, Pavlovian conditioning connects a substance with therapeutic effect and gustatory, tactile, visual, or contextual cues present in the situation because of repetition; hence these cues cause the same response that the substance does (4, 7). In the context of substance use, Pavlovian responses to these drug related cues may not only produce a similar pharmacological effect to that of the drug, but it has also been involved in the development of drug- opposite responses that can become conditioned to cues of initial drug onset and play a role in the development of both compensatory behaviors such as tolerance, where a compensatory response that reduces the initial drug effect when the substance is expected has been observed, or in the elicitation of withdrawal-like symptoms in cases in which addicted individuals are exposed to small doses of the drug they usually consume (8–10). Moreover, instrumental conditioning explains the relation between individuals’ response and consequences of their behavior (reward or punishment), so that the behavior will have a higher or lower probability of being repeated (11, 12). In addition, it was found that partial reinforcement produces a weaker placebo effect than continuous reinforcement and it is more resistant to extinction. This can be used in clinical practice to extend the improvement of the symptoms (2, 13).

Suggestion refers to the verbal information given to an individual which causes the activation of memories of previous experiences to generate expectancies about the results (2). With regard to social learning, Colloca and Benedetti (14) were the first authors who associated it with the placebo effect. Their study showed that when participants believed that other participants informed pain relief after the false activation of an electrode, they would feel the same therapeutic effect following their electrode activation. It is not necessary to hide that a placebo is being administered since it has been demonstrated that it also produces a significant enhancement (15–18).

Other aspects than can influence the placebo effect are personality factors, having found that characteristics like optimism (2, 19) and empathy (14, 20, 21) are associated with a higher placebo effect.

Numerous brain areas are related with placebo effect. Most studies have focused on brain areas implicated in the placebo effect in pain situations. Studies have shown that different regions show a lower activation measured by functional magnetic resonance imaging (fMRI) during placebo effect in pain response: thalamus, basal ganglia, somatosensory cortex, anterior cingulate cortex, insula, amygdala, and right lateral prefrontal cortex (2). Eippert et al. (22) observed a reduced sign by fMRI in the ipsilateral dorsal horn during the analgesia produced by placebo. It’s important to underlie the role of neurochemical processes in placebo effect. This response has been associated with an increase in endorphins, dopamine and opioid neurotransmission (23). Cannabinoid and cholecystokinin systems have been involved in the increase and reduction of placebo analgesia respectively (2). The understanding of the mechanisms underlying placebo effect is relevant to enhance the effectiveness of pharmacological and psychological treatments (2).

Traditionally, placebo effect has been examined in pain studies, however, in recent years it has received a growing interest due to its possible implication in the use of psychoactive substances. Some licit psychoactive substances are nicotine, caffeine, and alcohol. Nicotine is a highly addictive alkaloid present mainly in the tobacco plant and it has both stimulant (increasing cognitive performance) and depressant effects, alleviating pain, anxiety, and depression (24, 25). Caffeine is an alkaloid which can be found in coffee and cocoa beans, kola nuts, and tea leaves. It stimulates the central nervous system increasing alertness and attention, and reduces sleepiness (26, 27). Alcohol is a substance with both stimulant, causing talkativeness and euphoria at low doses (28) and sedative-hypnotic properties, provoking drowsiness or respiratory depression, at higher doses (29). When prolonged consumption of a drug is discontinued or rapidly reduced, a set of signs and symptoms can occur, this being called withdrawal syndrome (30). In the abstinence period, craving exerts an important role because it may lead to relapse. Craving has been defined as a subjective, motivational, and emotional state of desire to consume a substance when it is not available or during abstinence (31).

The effects of psychoactive substance consumption on craving and cognitive performance are widely known. However, the effect of the experience of having consumed a psychoactive substance as caffeine on cognitive performance is not clear (32–34). Furthermore, the effect of expectancies on craving has begun to be studied recently (35), and there is scarce evidence of its action on craving for alcohol, caffeine, or nicotine.

Craving and cognitive performance may be related when individuals, as regular consumers, know the behavioral and cognitive effects of a substance. Intense craving may affect cognitive performance, and in cases of substance dependent individuals, exposure to the substance related cues can elicit craving and impair performance on cognitive tasks. An attentional bias, where increased attentional priority is given to the substance cues presented while performing the task reduces individual’s overall cognitive resources for task performance, has been described consistently in individuals dependent on nicotine and alcohol (36, 37). But also, compensatory behaviors may develop, and under certain circumstances experienced consumers may develop compensatory behaviors that counteract the expected impairment caused by the consumption of the substance, as Fillmore and Vogel-Sprott (38) found, relating social drinking history, behavioral tolerance and the expectation of alcohol. These authors reported that experienced drinkers showed a drug-opposite improvement in performance after receiving a placebo when they were expecting alcohol. Results suggests that a compensatory response to the expectation of alcohol may participate in the greater behavioral tolerance observed in the more experienced consumers.

Studies indicate that consumption of placebo alcohol promotes craving and increase ad libitum alcohol intake (39–41). This effect seems not to be limited to alcohol priming, since placebo alcohol impairs inhibitory control (42). Motor performance is also affected by placebo alcohol (43–45). The anticipated effects of alcohol may depend on individual differences in alcohol-outcome expectancies. Thus, impairments in inhibitory control and motor performance following placebo-alcohol correlate with expectation of alcohol-induced cognitive impairment (42–45). Interestingly, Fillmore et al. (44) found that participants induced to expect alcohol-induced impairment on a rotor task showed a better performance than participants induced to expect alcohol-induced improvement. As the authors suggest, this may be a result of individuals intent to compensate for the expected impairment of alcohol. Compensatory mechanisms have also been suggested to underlie a drug-opposite improvement in performance when experienced participants expected alcohol but received a placebo. This improvement was not observed in nonexperienced participants, which suggests that a compensatory response to the expectation of alcohol contributes to the behavioral tolerance shown by experienced alcohol consumers (38).

In order to examine the influence of placebo effect on craving and cognitive performance outcomes in alcohol, caffeine, and nicotine consumers, a systematic review of the studies published in the last decade is now conducted.

## Methods

The selection of bibliographic search and critical assessment of pertinent studies related to our topic was performed according to the PRISMA guidelines (46).

### Research Strategies

A systematic analysis of the scientific literature was carried out by selecting articles published in PubMed, Web of Science, and Scopus databases. The search was conducted between December 15, 2019 and March 3, 2020.

Restrictions were made, limiting the research to papers published from 2009 until 2019. This temporal criterion was established because of the recent interest in this field, and the last ten years is the period when most studies involving the target variables were expected to have been published. The articles published before 2009 were removed through timing filters on databases.

Manually selected articles were added to the final list after reading the full text of the selected papers, mostly because the descriptor “placebo effect” could exclude some relevant papers, the descriptor “placebo” was not used because the result of the search would include a very large amount of article where this term is used for the design of a study, the substance used or its effects, not only the placebo effect. So that we decided to complete the databases research with a manual one.

The research strategy used the following terms:

- PubMed: (“placebo effect”[Title/Abstract] OR “placebo response”[Title/Abstract]) AND (expectancy[Title/Abstract] OR conditioning[Title/Abstract]) AND (alcohol[Title/Abstract] OR caffeine[Title/Abstract] OR tobacco[Title/Abstract]).
- Web of science: TS= (“placebo effect” OR “placebo response”) AND TS= (expectancy OR conditioning) AND TS=(alcohol OR caffeine OR nicotine OR tobacco).
- Scopus: (TITLE-ABS-KEY (“placebo effect”) OR TITLE-ABS-KEY (“placebo response”) AND TITLE-ABS-KEY (expectancy) OR TITLE-ABS-KEY (conditioning) AND TITLE-ABS-KEY (alcohol) OR TITLE-ABS-KEY (caffeine) OR TITLE-ABS-KEY (nicotine) OR TITLE-ABS-KEY (tobacco)).

### Eligibility Criteria

From the preliminary list of potential papers found by systematic analysis, only studies which fulfilled the following criteria were selected:

1. Papers whose topic was placebo effect on craving or cognitive performance in nicotine, caffeine, or alcohol.
2. Papers written in English.
3. Studies with N ≥ 30.
4. Experimental studies.
5. Papers published between 2009 and 2019.

## Results

### Papers Selection Process: Flow Diagram

**Figure 1:**
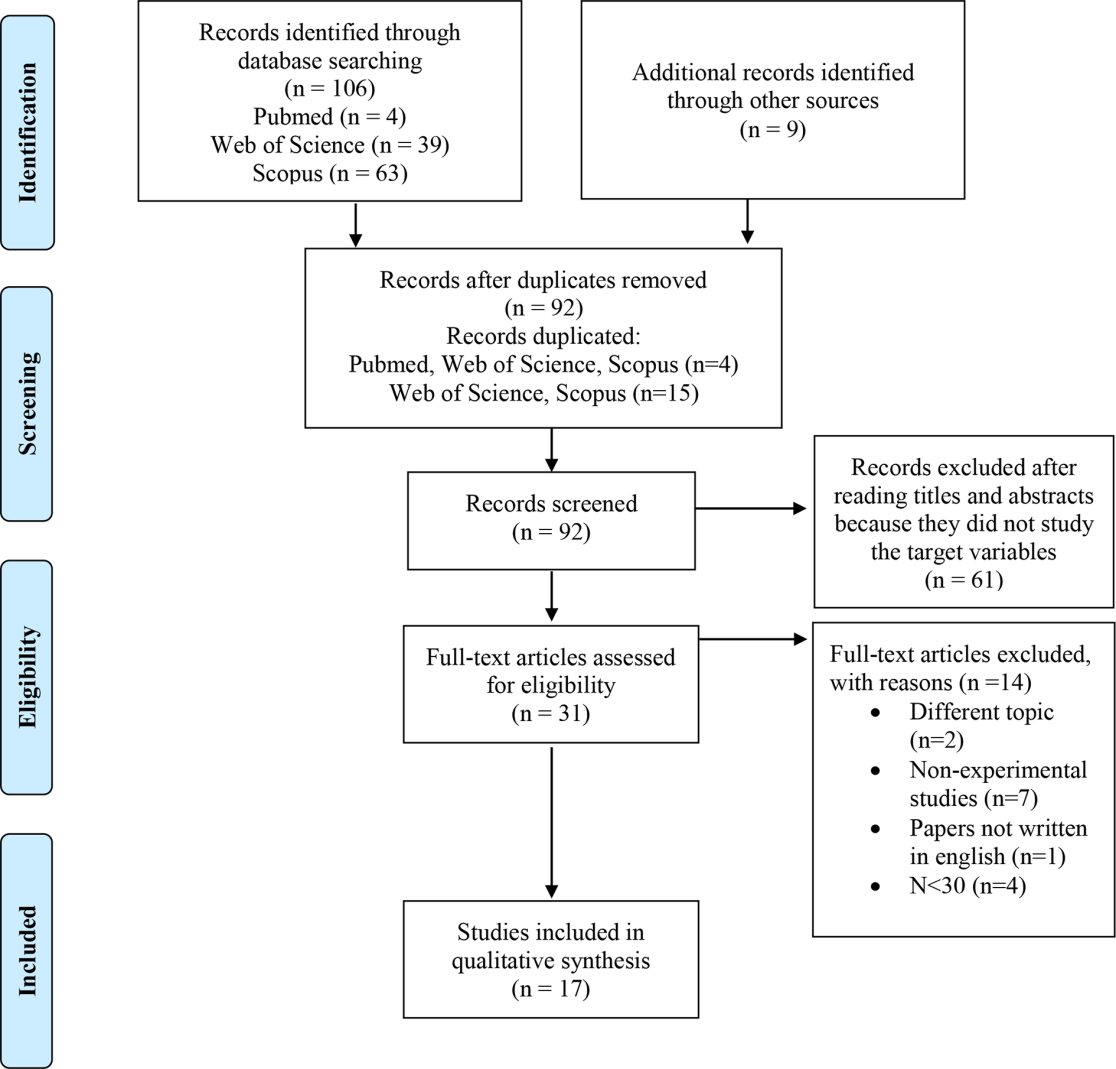
Flow chart for paper selection.

The number of preliminary selected papers was 115, 106 records identified through database searching and 9 additional records identified through a manual search. Twenty-three articles were duplicated and consequently removed. There were 92 potential papers, however, after reading tittles and abstracts, 61 papers were excluded because they did not study the variables to be analyzed in this systematic review. After reading the remaining 31 articles, 14 papers were eliminated following the inclusion criteria. Therefore, 17 articles were finally selected for the systematic review. **Figure 1** shows the process of identification, selection, eligibility, and inclusion of the papers in a flow diagram/chart.

### Description of Studies Characteristics

**Table 1** describes the main characteristics of the 17 studies selected, including authors and year of publication, aim of the study, participants, and variables and measures used.

**Table 1:** Characteristics of studies.

Authors and year of publication	Aim of the study	Participants	Variables and measures used
Harrell and Juliano (47)	To analyze the effect of caffeine expectancies on cognitive performance.	N=60 participants who consumed between 200 and 800 mg of caffeine per day and drank coffee at least five times per week. 68% women. 32% men.	Cognitive performance (RVIP and finger tapping task)
Leeman et al. (48)	Comparison of craving in placebo and alcohol conditions and to study if craving predicts the *ad libitum* consumption after a placebo but not after alcohol administration.	N=174 participants. Men consumed at least 5 drinks a day during the last month and women at least four drinks. 50%, 30% women. 49%, 70% men.	Craving (a single item in a visual analogical scale) *Ad libitum* alcohol consumption (BAC).
Perkins et al. (49)	To examine the effects of expectancies on smoking response *via* nasal spray.	N=93 participants who smoked at least 10 cigarettes daily. 45%, 36% women. 54%, 64% men.	Craving (QSU-B) Nicotine withdrawal (Scale) Reward value (the question ‘How much do you like the spray?’)
Gilbertson et al. (50)	To study the influence of alcohol expectancies on cognitive performance.	N=30 participants who are older social drinkers. 50% women. 50% men.	Cognitive performance (Posner paradigm) Subjective intoxication and impairment (self-informed measures)
Dawkins et al. (51)	To explore if caffeine expectancies influence attention, mood, and reward responsivity.	N=88 participants who drink two or more cups of coffee per day. 50% women. 50% men.	Attention (Stroop Task) Reward responsivity (CARROT)
Juliano et al. (52)	To explore the effect of nicotine and nicotine expectancies on attention, smoking urge, mood, and cigarette ratings.	N=148 participants who smoked at least 10 cigarettes daily. 44%, 59% women. 55%, 41% men.	Smoking outcome expectancies (SCQ-A) Craving (a self-report) Attention task (RVIP)
Harrell and Juliano (53)	To study the influence of nicotine expectancies on craving and cognitive performance.	N=80 participants who smoke 6–40 cigarettes per day. 30% women 70% men.	Cognitive task (RVIP) Withdrawal scale (MNWS) Craving (Questionnaire of Smoking Urges)
Bombeke et al. (54)	To examine alcohol effects on post-error adjustments focusing on PES, PERI and PIA.	N=45 participants who drink 1.8–3.5 drinks per day. 100% men.	Congruency task (Stroop task)
Darredeau et al. (55)	To explore the role of nicotine expectancies in subjective and behavioral variables.	N=60 dependent and nondependent smokers. 50% women. 50% men.	Craving (QSU-B)
Weimer et al. (56)	To study the effect of nicotine expectancies on reaction time.	N=64 participants who are smokers and nonsmokers. 50% women. 50% men.	Craving (VAS) Neurocognitive task (PGNG)
Schlagintweit et al. (57)	To explore the impact of 4mg of nicotine in mood, craving, and heart rate.	N=70 dependent smokers. 48%, 57% women. 51%, 43% men.	Craving (VAS, QSU-B)
Dömötör et al. (58)	To study the impact of 5mg of caffeine and the influence of caffeine expectancies on HR, SBP/DBP, HRV RT, and subjective variables.	N=107 participants. 60%, 70% women. 39%, 30% men.	Response expectancies (SRQ) Reaction time (PsychLabWin v1.1 software)
Christiansen et al. (42)	To examine the effect of alcohol expectancies on craving and inhibitory control.	N=32 nondependent drinkers. 65%, 63% women. 34%, 37% men.	Craving (DAQ) Alcohol outcome expectancy (AOES) Inhibitory control (PGNG)
Mills et al. (59)	To analyze if caffeine expectancies reduce caffeine withdrawal and craving symptoms.	N=89 participants who drink at least three cups of coffee every weekday. 67%, 42% women. 32%, 58% men.	Neurocognitive task (RVIP)
Robinson et al. (60)	To examine the effects of nicotine administration and nicotine expectancies on attentional bias to smoking affective cues.	N=51 participants who smoked at least 10 cigarettes every day. 47%, 06% women. 52%, 94% men.	Picture Distracter Stimuli (Pleasant, unpleasant, neutral, and cigarette-related pictures from IAPS); Cigarette-related pictures from ISIS and developed in their lab) RT (RVIP-CED)
Knibb et al. (61)	To explore the influence of the belief that alcohol can impair behavioral control in alcohol priming effect and alcohol induced impairments on inhibitory control.	Study 1: 81 participants 45%, 68% women. 54%, 32% men. Study 2: 82 participants 64%, 63% women. 35%, 37% men.	Craving (DAQ) Inhibitory control and RT (SST)
Palmer and Brandon (62)	To examine the effects of nicotine and expectancies on craving to smoke and vape.	N=130 participants who smoked e-cigarette or with history of smoking 1 cigarette per day. 38% women. 62% men.	Craving (QSU-B)

AOES, Alcohol Outcome Expectancies Scale; BAC, Blood Alcohol Concentration; CARROT, Card Arranging Reward Responsivity Objective Test; CWSQ, Caffeine Withdrawal Symptom Questionnaire; DAQ, Desires for Alcohol Questionnaire; IAPS, International Affective Picture System; ISIS, International Smoking Image Series; MNWS, The Minnesota Nicotine Withdrawal Scale; PGNG, Parametric Go/No-Go task; QSU-B, Smoking Urges-Brief; RVIP, Rapid Visual Information Processing task; RVIP-CED, Rapid Visual Information Processing Task with Central Emotional Distracters; SCQ-A, Smoking Consequences Questionnaire-Adult; SRQ, Self-Informed Questionnaire; SST, Stop-Signal Task; VAS, Visual Analog Scale.

These articles were published between 2009 and 2019. They were conducted in different regions: eight papers in the United States of America, three in Europe, two papers from Canada, three papers from the United Kingdom, and one from Australia.

Regarding the substance studied in the research, five papers studied alcohol (42, 48, 50, 54, 61), four papers caffeine (47, 51, 58, 59), and eight papers nicotine (49, 52, 53, 55–57, 60, 62).

With regard to the sample of participants, there were participants between 18 and 59 years in alcohol papers, between 18 and 47 years in caffeine papers, and between 17.8 and 65 years in nicotine studies. There were more women than men in six papers, more men than women in seven studies, the same number of women and men in four papers, and only men in one paper. Participants were recruited from university students in eight papers, from other populations in seven papers and including both university students and other populations in three papers.

Regarding craving, it was measured by the Desires for Alcohol Questionnaire (DAQ), Caffeine Withdrawal Symptom Questionnaire (CWSQ), the questionnaire of Smoking Urges-Part B (QSU-B), the Minnesota Nicotine Withdrawal Scale (MNWS), the questionnaire of smoking urges, a self-report, and a single item visual analog scale. Cognitive performance was measured by the Parametric Go/No-Go task (PGNG), the Rapid Visual Information Processing Task (RVIP), the Rapid Visual Information Processing Task with Central Emotional Distracters (RVIP-CED), Stroop task, and a reaction time task in a PsychLabWin v1.1 software.

With regard to the last time participants consumed the substance before the study, in most studies participants were asked to abstain to consume the drug except in the study of Leeman, Corbin & Fromme (48). Dawkins et al. (51) and Dömötör et al. (58) asked it but they did not explicit if they had taken measures to confirm it. In alcohol studies abstinence was confirmed by BrAc (50) and BAC (47, 54, 61). In caffeine studies abstinence was confirmed by a saliva sample (47, 59). In nicotine studies abstinence was confirmed by carbon monoxide sample (49, 52, 53, 55, 57, 60, 62).

The exact placebo manipulation used in alcohol, caffeine and nicotine studies was as follows.

#### Alcohol Studies

The alcohol drink used in alcohol condition was vodka except in the study of Gilberston et al. (50) where it was used 100% medical grade alcohol and in the study of Christiansen et al. (42) where alcohol was not served. In Leeman, Corbin & Fromme’s study (48) drink volumes were adjusted according to weight and gender of each individual to reach a target BAC of 0.06 g; in the study of Gilberston et al. (50) it contained 1/3 parts of alcohol and three parts; in the study of Bombeke et al. (54) beverage contained 0.55 g of alcohol; in the study of Knibb et al. (61) alcohol drinks contained 0.5g of vodka per kg of body weight; in the study of Gilberston et al. (50) alcohol was administered to achieve a BrAC level of 40 mg/100 ml during the task. In all studies with alcohol condition, alcohol beverage was mixed with another drink (48, 50, 54, 61). Placebo beverage was made of flat tonic water mixed with Cherry 7-Up and lime juice in Leeman, Corbin & Fromme’s study (48), ice-cold noncaffeinated lime soda in the study of Gilberston et al. (50) or lemonade (42, 61). To provide sensory cues, the glasses used were rimmed with vodka (42, 48, 61) and alcohol on the surface (42, 48, 50) to provide sensory cues. In addition, in placebo group of the study of Leeman, Corbin & Fromme (48) flat tonic water was poured from a vodka bottle in direct sight of the participants and the bar was cleaned with alcohol. In Bombeke et al’s study (54) the presence of alcohol was hidden with green peppermint syrup and covering the cups, so the participants had to drink with a reed. The study of Christiansen et al. (42) and Knibb et al. (61) had a taste test and the drinks served were nonalcoholic beer or orange flavored drink (nonalcoholic beverage too) and alcoholized beer (Skol 2.8% ABV) or Skol with 10ml of lemonade in the latter. Christiansen et al. (42) included a control group whose drink was water.

#### Caffeine Studies

In caffeine studies decaffeinated coffee was served in placebo groups to provoke sensory cues (51, 58, 59). Caffeine group in the study of Dawkins et al. (51) a one teaspoon of caffeine was provided, in Dömötör et al.’s (58) caffeinated drinks had 5 mg/kg and in Mills et al.’s (59) it was served ≤ 4 mg per cap. In Dömötör et al. (58) it was a natural history group who drank water.

#### Nicotine Studies

In placebo group different placebos have been used, a placebo spray which did not contain nicotine but produced sensory irritating effects as those of Nicotrol was used (49), a placebo cigarette with no more than 0.05 mg nicotine and 10 mg of tar (52, 53, 55), a chewing gum with no nicotine (56), a lozenge which did not contain nicotine (57) or e-cigarettes contained no nicotine (62). In nicotine group it was served a nicotine cigarette contained 0.6 mg of nicotine and 10 mg of tar (52, 53, 55, 60) a chewing gum with 2 mg of nicotine (56), a lozenge which contained 4 mg of nicotine or e-cigarettes contained 12mg/l of nicotine (62). In addition, in Juliano et al. (52) and Harrell & Juliano’s studies (53) participants were given a menthol cigarette or nonmenthol cigarette depending on their usual smoking preferences.

With regard to the strength of the beliefs of the researchers’ instructions, participants in alcohol studies were asked to rate the strength of their drinks except in Leeman, Corbin & Fromme’s study (48). After the consumption, they were asked to answer a self-report questionnaire about the perception of being intoxicated (50) or a questionnaire about their beliefs of having consumed an alcohol beverage (54) or estimate how many standard UK units of alcohol had drunk in the study (42, 61). Those in caffeine studies were asked to rate it too. After consuming the beverage, they answered questions about drug resistance, coffee dimensions, caffeine content of the beverage (47), if they had suspected the deception (51, 59) postexperimental analysis were conducted (58). Participants in nicotine studies were asked to rate the strength of their cigarettes, e-cigarettes, chewing gum, or spray, except in the study of Schlagintweit et al. (57). Participants had to answer if the spray contained nicotine or were asked to estimate the nicotine dose received (49, 62) or to answer questions about nicotine sensations (52), to complete a scale of nicotine effect on cognitive performance (53, 56) or to fill a questionnaire to evaluate the deception (49, 51, 60).

### Description of the Principal Results

**Table 2** shows the main outcomes of the papers analyzed.

**Table 2:** Description of the main outcomes.

Authors and year	Design	Statistical analysis	Results
Harrell and Juliano (47)	Double-blind, between-subjects design	ANOVA Chi-squares *t* tests	Participants in caffeine condition had a bigger improvement in reaction time, hits and sensitivity on RVIP and taps per second on the finger tapping task. Participants in told impair/given placebo condition reduced their reaction time and increased their hits and sensitivity on RVIP. However, those in told enhance/given placebo increased their reaction time and reduced their hits and sensitivity on RVIP. Participants in told impair/given caffeine condition had less withdrawal alleviation than those in told enhance/given caffeine condition, with no effect of the expectancy manipulation.
Leeman et al. (48)	Placebo controlled design	ANOVA Hierarchic and multiple regression analyses. Multi-level models	Trait disinhibition, but not avoidance/inhibition of harm, predicted craving. Trait disinhibition predicted *ad libitum* consumption and craving predicted trait disinhibition, *ad libitum* consumption, and the other variables entered into the model. Only the effect of trait disinhibition on *ad libitum* consumption was not significant when craving was included in the model.
Perkins et al. (49)	2 × 2 balanced placebo design	ANOVA	Craving was decreased after nasal spray exposure by actual nicotine and nicotine expectancy.
Gilbertson et al. (50)	Double-blind, placebo-controlled design	ANOVA Bonferroni correction	There were differences in RT showing that alcohol group and placebo group had similar RT in the following measures: Correctly Cued: Correct; Incorrectly Cued: Correct; Neutrally Cued: Correct; and Neutrally Cued: Wrong. Alcohol group was more delayed in Correctly Cued: Wrong, and Incorrectly Cued: Wrong conditions than nonalcohol group and placebo group. Participants in told/given alcohol showed more impairment in self-informed than other groups.
Dawkins et al. (51)	Double-blind, between-subjects design	ANOVA *t-*tests	Caffeine caused better accuracy on incongruent, but not congruent, trial, especially in Told Caffeine group. This group showed shorter RT on congruent trials. Card sorting was significantly faster on the rewarded trial in Caffeine group and in Told Caffeine group. Told Caffeine group was faster in nonrewarded and rewarded trials and showed higher reward responsivity.
Juliano et al. (52)	Balanced placebo design	ANOVA ANCOVA	Participants in given nicotine condition exhibited shorter RT and greater sensitivity on the task and number of hits. Participants in told nicotine condition showed fewer false alarms. Participants in told/non given nicotine group informed lower smoking. Craving was reduced in nicotine group and nicotine expectancies group.
Harrell and Juliano (53)	2 × 2 between-subjects factorial design	ANCOVA Chi squares test	Participants in nicotine condition had a higher sensitivity and a bigger number of hits. Participants in “told enhance” condition informed lower craving. Participants who were given a nicotine cigarette informed lower craving. Participants showed longer withdrawal and higher urge rating on experimental session. Participants had shorter withdrawal after cigarette consumption in the nicotine condition.
Bombeke et al. (54)	Double-blind, between-subjects design	ANOVA	Participants in told/given placebo group were slower than those in told/given alcohol and told/non given alcohol group.
Darredeau et al. (55)	Mixed, balanced placebo design	ANOVA	Men in told/given nicotine, women in told/non given nicotine, and dependent smokers tried harder to earn puffs and higher amounts of self-administration. Nondependent men in told/non given placebo were slower to earn the first puff. Craving was decreased after cigarette sampling when participants were told it was nicotine-free and increased when nondependent women were told cigarettes contained nicotine.
Weimer et al. (56)	Double-blind, balanced placebo Design	ANOVA *t* tests	In told nicotine condition, nonsmoking women showed longer RT at all levels, smoking women had shorter RT at all levels, nonsmoking men showed slower RT at level 1 and faster RT at levels 2 and 3, and smoking men had shorter RT at level 2 and slower RT at levels 1 and 3.
Schlagintweit et al. (57)	Balanced-placebo Design	Mixed models method	A lower craving was found in told/given nicotine condition and following lozenge consumption and neutral cue. A higher withdrawal-related craving was produced in female following the smoking cue than after lozenge consumption and the neutral cue.
Dömötör et al. (58)	Double-blind, placebo-controlled design	Multiple linear regression analysis	Having consumed caffeine was a significant predictor at T2 values of SRQ, but not of RT. Response expectancy score was a significant predictor of SRQ score at T2. The impact of baseline SRQ score and actual caffeine intake was significant in the first equation. In the final equation, both of these variables remained significant and response expectancy score also reached significance level.
Christiansen et al. (42)	Within- Subjects Design	ANOVA *t* test Pearson’s correlation	Participants in placebo condition showed increased craving compared to control condition. Participants in placebo condition committed a higher number of no-go errors than participants in control condition. There were two positive correlations in placebo condition: one between no-go errors and expectancies of impaired cognitive performance, and the other between craving changes and positive and negative alcohol outcome expectancies.
Mills et al. (59)	2×2×(2) mixed design	ANOVA Simple linear regression analysis	CWSQ scores experienced a bigger decrease in participants in Told Caffeine condition that in Told Decaffeinate condition from pre- to post-beverage Specifically, it was showed a greater reduction in craving, decreased alertness and difficulty concentrating, drowsiness and fatigue and flu-like feelings. There was a significant time effect on CWSQ, systolic blood pressure and the RVIP false alarm rate, finding lower scores from pre-to post-beverage. There was a significant time effect on RVIP accuracy, finding bigger scores from pre- to post-beverage. The strength of caffeine expectancies significantly predicted the magnitude of the reduction in both total CWSQ score and craving.
Robinson et al. (60)	Within-subjects balanced placebo design	ANOVA *Post hoc* pairwise tests of simple effects	Smokers in told nicotine condition and those in given nicotine condition showed higher scores on Craving Reduction Scale of modified Cigarette Evaluation Questionnaire. Smokers in given nicotine condition got faster RT, less conservative response bias, greater sensitivity and accuracy, and higher hit and false positive rates.
Knibb et al. (61)	Mixed design with a within-subject factor of drink and a between- subject factor of condition	*t-*test ANOVA Chi-square	In both studies, participants in alcohol group had higher craving and felt less able to control their drinking. In study 1, participants in alcohol group felt more able to control drinking behavior and had greater inhibition errors than in the average condition. In study 2, craving was increased from baseline to post-drink and from baseline to the end of the session in alcohol and placebo group. Participants had greater SSRT after alcohol consumption in experimental condition. Increased inhibition errors were found after alcohol consumption.
Palmer and Brandon (62)	Balanced placebo design	ANOVA *t*-test	The estimation of a higher nicotine dose was associated to greater cigarette craving reduction. The estimation of nicotine dose was not associated to a reduction on e-cigarette craving. Participants in told nicotine condition showed a greater smoking craving reduction than those in nonnicotine condition. Participants in told nicotine condition showed greater reductions in vaping craving. Participants in told nicotine condition who received nicotine e-cigarette showed significantly greater reductions in craving to vape than those who belonged to other groups.

ANCOVA, Analysis of covariance; ANOVA, Analysis of variance; CWSQ, Caffeine Withdrawal Symptom Questionnaire; RT, Reaction Time; RVIP, Rapid Visual Information Processing task; SRQ, Self-informed questionnaire; SSRT, Stop signal reaction time

### The Effect of Expectancies on Craving

#### Alcohol

Regarding alcohol expectancies on craving, Leeman et al. (48) informed that alcohol expectancies caused similar levels of craving even when alcohol had not been consumed. Craving produced following a placebo consumption predicted ad libitum alcohol consumption. Ad libitum consumption in placebo situations was positively correlated to trait disinhibition and negatively correlated to harm avoidance/inhibition. Christiansen et al. (42) described that alcohol outcome expectancy increased craving and this was positively associated with expectancies of tension reduction and social facilitation and worst social, cognitive, and emotional performance. Knibb et al. (61) found that alcohol expectancies produced an increase of craving in alcohol and placebo condition, but this was bigger in alcohol group. In addition, there was not any difference between both conditions in ad libitum consumption.

Therefore, alcohol consumption increased craving and studies have demonstrated that alcohol expectancies in placebo condition produce an enhancement in craving too. Nevertheless, it is not clear if the intensity of craving and the frequency of ad libitum consumption is similar of both conditions, or higher on alcohol condition or in placebo condition (42, 48, 61).

#### Caffeine

As for caffeine expectancies on craving, Mills et al. (59) found that participants who believed they had consumed caffeine showed lower scores in total Caffeine Withdrawal Symptom Questionnaire (CWSQ) and they were less motivated to consume coffee. Participants who had not consumed caffeine showed a decrease in withdrawal symptoms too, which was explained by the authors by classical conditioning.

Thus, caffeine expectancies produced a craving reduction (59).

#### Nicotine

Concerning the effect of nicotine expectancies on craving, Perkins et al. (49) informed that receiving nicotine or expecting it caused a decreased craving after the spray dose. Juliano et al. (52) found that receiving nicotine or expecting it attenuated smoking urges. Participants who expected nicotine had greater rewarding effects than those who expected placebo. Participants who received a nicotine cigarette spent more time smoking than those who received placebo cigarette. Participants who expected nicotine and received it took more puffs than those who expected placebo, but participants who expected nicotine but received placebo took fewer puffs than those who expected placebo. Harrell and Juliano (53) informed that smokers who were told that nicotine cigarettes would improve performance informed craving reduction, psychological reward and a greater motivation to perform well the task, independently of the nicotine content. Although, craving reduction was bigger in nicotine condition. Smokers who were told that nicotine cigarette would impair their performance did not feel these benefits. Darredeau et al. (55) found that the expectancy of receiving nicotine cigarette made participants to try harder to earn cigarette puffs and self-administered them with a higher frequency. Participants who were told that the cigarette did not contain nicotine showed a post-sampling decrease in craving. When women believed that they had received nicotine cigarettes, postsampling craving was increased across every cigarette conditions; when men believed that they had received nonnicotine cigarette they delayed self-administration of nicotine cigarette. Schlagintweit et al. (57) found that nicotine expectancies reduced craving even when nonnicotine lozenge had been consumed. Craving was increased when participants were exposed to smoking cues and this increase was higher in women than in men. Palmer and Brandon (62) informed that the expectancy of receiving a nicotine e-cigarette reduced significantly smoking and vaping craving. The estimation of a higher nicotine dose was associated with greater smoking craving reduction.

Hence, most studies (49, 52, 55, 57, 62) have demonstrated that expectancies of consuming nicotine reduce craving even when a placebo has been received. Only the results of Harrell and Juliano (53) differ since they found that being informed that nicotine cigarettes would improve performance showed a craving reduction, independently of the cigarette content, but this decrease was not found when participants were informed that nicotine cigarette would impair performance. In addition, Darredeau et al. (55) and Schlagintweit et al. (57) have informed that gender may have an influence on the craving response.

### The Effect of Expectancies on Cognitive Performance

#### Alcohol

In relation to alcohol expectancies on cognitive performance, Gilbertson et al. (50) found that the expectancy of having consumed alcohol slowed reaction time on Posner paradigm even when alcohol was not consumed. However, accuracy on this paradigm was not affected by alcohol expectancies. Bombeke et al. (54) informed that performance of participants who believe they were intoxicated was similar to performance of those intoxicated because both alcohol and placebo condition had a smaller post-error slowing than control group. Christiansen et al. (42) found that alcohol outcome expectancy can cause significant impairments on inhibitory control. Knibb et al. (61) described in their study 1 that participants who believed that they had average self-control got higher scores of inhibitory errors when alcohol beverage was consumed. In study 2, alcohol expectancy did not cause any difference in go reaction times between alcohol condition and placebo condition, however, inhibition errors were greater following alcohol consumption.

Studies show that alcohol expectancies did not cause any difference (61) or slowed reaction time (50) and reduced post-error slowing in alcohol and placebo condition (54) but did not affect accuracy (50). Christiansen et al. (42) informed that alcohol expectancies can cause impairments on inhibitory control, although Knibb et al. (61) reported increased inhibitor errors after alcohol consumption. Bombeke et al. (54) observed that participants who expected to consume alcohol showed smaller post-error slowing in placebo and alcohol conditions.

#### Caffeine

As for caffeine expectancies on cognitive performance, Harrell and Juliano (47) informed that only participants in caffeine group showed an enhancement in reaction time and accuracy. Participants who were given placebo and expected that caffeine would impair their performance performed better than those who expected that caffeine would improve it. Dawkins et al. (51) found that caffeine did not influence reaction time but it enhanced accuracy on the Stroop task, particularly on incongruent trials. However, expectancies of having consumed caffeine enhanced accuracy and reaction time on congruent and incongruent trials. Having consumed caffeine or the expectation of caffeine consumption increased speed on the Card Arranging Reward Responsivity Objective Test (CARROT). Dömötör et al. (58) informed that caffeine consumption or the expectancy of receiving it did not influence participant’s reaction time.

Harrell and Juliano (47) found that receiving caffeine improved reaction time and accuracy but the expectancy of receiving it or the placebo did not. Dawkins et al. (51) informed that caffeine expectancies enhanced reaction time in placebo group and accuracy in caffeine and placebo condition in Stroop task and both groups had better reaction time in CARROT task, however, Dömötör et al. (58) did not find any significant effect.

#### Nicotine

Regarding nicotine expectancies on cognitive performance, Juliano et al. (52) informed that participants who were given nicotine cigarettes showed faster reaction time and greater accuracy and sensitivity on the RVIP. Participants who expected receiving a placebo had a greater number of false alarms than those who were expecting nicotine. Harrell and Juliano (53) found that nicotine administration caused a greater number of hits and sensitivity but it did not affect to reaction time on the RVIP. Told that nicotine enhance or impair performance had no effect on attention performance measured by the RVIP task. Weimer et al. (56) described that nicotine information caused a longer reaction time in nonsmoking women than placebo information. However, the same information caused a shorter reaction time in smoking women. Nicotine instruction caused a slower reaction time at level 1 in Go/No-Go task but faster reaction time at levels 2 and 3 in nonsmoking men, however, the same information caused faster reaction time at level 2 but slower reaction time at levels 1 and 3 in smoking men. Robinson et al. (60) found that after an acute nicotine deprivation, to receive nicotine or the expectancy of it decreased the distractibility of motivationally significant stimuli and promoted the recognition of motivationally significant stimuli on Rapid Visual Information Processing task with central Emotional Distracters (RVIP-CED).

Juliano et al. (52) informed that expecting to receive a placebo produced greater number of false alarms that expecting to receive nicotine, and Robinson et al. (60) found that the expectancy of receiving nicotine decreased the distractibility of motivationally significant stimuli and promoted the recognition of it. Harrell and Juliano (53) described that nicotine expectancies did not produce any effect on reaction time, number of responses or sensitivity. However, Weimer et al. (56) informed that nicotine expectancies caused a different response in function of smoking status and gender, finding an increase in the reaction time in nonsmoking women and a decrease in smoking women. Nicotine expectancies performed differently in smoking/nonsmoking men but the results were less clear.

## Discussion

This systematic review examines the impact of alcohol, caffeine, and nicotine expectancies on craving and cognitive performance. An influence of alcohol expectancies on craving has been found in several studies, showing that alcohol expectancies can cause an increased craving even when alcohol has not been consumed, although it is not clear if the craving intensity is similar in placebo and alcohol condition or bigger in one of them (42, 48, 61). Furthermore, a positive correlation between craving and expectancies of tension reduction, social facilitation and worse social, cognitive and emotional performance was found (42).

Findings suggest that the expectancy of having consumed caffeine can reduce craving and the symptoms associated with it (59).

Regarding the effect of nicotine expectancies on craving, it was found a reduction in craving when a placebo is administered by cigarettes, nasal spray or e-cigarettes (49, 52, 55, 57, 62). Only the results of Harrell and Juliano (53) are different because they found that being informed that nicotine cigarettes would improve performance showed a craving reduction independently of the cigarette content but this decrease was not found when participants are informed that nicotine cigarette would impair performance. Darredeau et al. (55) and Schlagintweit et al. (57) have informed that gender can influence on the craving response and these authors (57) described that when smoking cues are presented, craving is increased.

With respect to the effects of alcohol expectations on cognitive performance, it was not found an effect in reaction time by expectancies of having consumed alcohol in alcohol and placebo condition in Knibb et al.’s study (61) nor an effect on accuracy Gilbertson et al. (50). Nevertheless, these authors (50) found that it caused a slower reaction time even when alcohol was not consumed. When it is concerned to post-error slowing, performance of participants who believed they were intoxicated was similar to those of intoxicated participants (54). Christiansen et al. (42) showed that expectancies of alcohol effects can cause significant impairments on inhibitory control and, specifically, Knibb et al. (61) found that there was a poorer inhibitory control following alcohol consumption.

The role of caffeine on cognitive performance has not been clarified, thus, Harrell and Juliano (47) described that caffeine administration improved reaction time and accuracy but not to expect caffeine and receive a placebo. Dawkins et al. (51) informed that the expectancy of having consumed caffeine improved accuracy (Stroop task) and reaction time (Stroop and CARROT tasks) in placebo condition and accuracy (Stroop task) and reaction time (CARROT task). However, it did not influence participant’s reaction time on Dömötör et al.’s research (58).

The study of the effect of nicotine expectancies on cognitive performance has also shown mixed results. Harrell and Juliano (53) found that expecting nicotine had no effect on reaction time, number of responses or sensitivity but Robinson et al. (60) described that nicotine expectancy can improve the attentional filtering of distracting stimuli. Juliano et al. (52) informed that expecting a placebo cigarette caused a greater number of false alarms that expecting a nicotine one. Weimer and colleagues (56) informed that nicotine expectancies caused a different response in function of smoking status and gender, finding that it slowed reaction time in nonsmoking women and produced a faster reaction time in smoking women. Nicotine expectancies performed differently in smoking or nonsmoking men but the outcomes are unclear.

There are some limitations that need to be addressed after the fulfilment of this systematic review. First, the number of studies that could be included, which is only 17, thus, the findings of this review can be modified by future studies. Second, the sample was diverse, finding that some papers included a sample of dependent participants, nondependent participants or both of them. In line with this, individual differences regarding number of years of experience with the substance and development of compensatory behaviors should be considered. Third, in some studies craving was not assessed by a questionnaire statistically validated. Fourth, in the same sense, most caffeine and nicotine studies did not measure abstinence using objective tests, thus, participants could have misinformed recent consumption. Finally, while some studies were conducted in seminaturistic environments simulating a bar, others used laboratory settings, leading to a difficult integration of the results observed. Thus, in the context of substance use, previous experience with the substance may contribute to symptoms of withdrawal after the administration of a low dose of the substance (9). Under certain circumstances, environmental cues associated with the consumption of the substance, may induce drug-opposite conditioned effects that resembles symptoms of substance withdrawal, making experience with the substance and the context two very important variables that could contribute to the results observed. All these limitations should be considered in further studies for a better understanding of this topic. Strengths of the present review include the utilization of study review eligibility criteria which were applied by two independent reviewers and the establishing a minimum of 30 participants per study, which is required to create a normal distribution and draw significant conclusions.

In summary, the role of expectancies on craving has been delineated. Specifically, while alcohol beliefs can cause an increase in craving, although it’s not clear if they affect differently alcohol and placebo condition, caffeine and nicotine expectancies tend to reduce craving. As for the influence of expectancies on cognitive performance, it was found that alcohol expectancies cause similar reaction times and accuracy in alcohol and placebo conditions or slower reaction time when alcohol is not consumed. Expectancies may impair inhibitory control, especially when alcohol has been consumed, and cause similar post-error slowing. Regarding the influence of caffeine expectancies on cognitive performance, research has not clarified its effects upon reaction time, however, it has been shown that accuracy is improved. Nicotine expectancies improve the attentional filtering of distracting stimuli. These results show that subjective and behavioral consequences of drug consumption are influenced by the expectancies of the effects of that substance. This has important implications because it suggests that expectancies may be considered a suitable target for treatment of substance use disorders. Specifically, it might be interesting in nicotine and caffeine use disorders. Individual differences in alcohol, nicotine and caffeine outcome expectancies may affect reactivity to the anticipated effects of the drug and consumption. The anticipated effects of these substances on craving and expectancies may thus rely on a combination of both pharmacological and individual expectancies of the effects of this substance, which should also be considered for future studies. Therefore, the results reported in thi  systematic review may be of interest to both clinicians and researches.

## Data Availability Statement

The original contributions presented in the study are included in the article/supplementary material; further inquiries can be directed to the corresponding author.

## Author Contributions

All authors contributed to the article and approved the submitted version.

## Funding

Funding for publishing this manuscript is provided by Consejería de Economía, Innovación, Ciencia y Empleo, Junta de Andalucía, Spain, Research Group CTS-195 Psicofarmacología Experimental, and from Grants for Open Access Publication from University of Málaga.

## Conflict of Interest

The authors declare that the research was conducted in the absence of any commercial or financial relationships that could be construed as a potential conflict of interest.
